# A Comb-Chain Cross-Linker-Based Network Solid Polymer Electrolyte for All-Solid-State Sodium-Metal Batteries

**DOI:** 10.1021/acsaem.5c02367

**Published:** 2025-09-11

**Authors:** William R. Fullerton, Haoruo Liu, David N. Agyeman-Budu, Jintao Fu, Mohamed H. Hassan, Mark C. Staub, Eric Detsi, Johanna Nelson Weker, Christopher Y. Li

**Affiliations:** † Department of Materials Science and Engineering, 6527 Drexel University, Philadelphia, Pennsylvania 19104, United States; ‡ Department of Materials Science and Engineering, 6572 University of Pennsylvania, Philadelphia, Pennsylvania 19104, United States; § Stanford Synchrotron Radiation Lightsource, 497525 SLAC National Accelerator Laboratory, Menlo Park, California 94025, United States; ∥ 316961 TA Instruments, New Castle, Delaware 19720, United States

**Keywords:** sodium-metal batteries, solid-state batteries, solid polymer electrolytes, network solid polymer electrolytes, network polymer catholytes

## Abstract

All-solid-state sodium-metal batteries (SMBs) utilizing solid polymer electrolytes (SPEs) have gained considerable research interest due to the potentially enhanced safety, lower cost, and sustainable sodium supply compared to lithium metal. However, sodium’s high reactivity makes it prone to dendrite and orphaned metal formation, reducing its capacity and efficiency. In this work, we report a comb-chain cross-linker-based network SPE for all-solid-state SMBs. The high-functionality macromolecular cross-linker offers excellent overall mechanical properties of the SPE. The polymer network exhibited an impressive elongation at break of 181% and a high toughness of 1.6 MJ m^–3^. These excellent mechanical properties, combined with good ionic conductivity and processability, enable ultrathin SPE separators and contribute to the superb dendrite resistance and full cell performance of the SPE. Na|SPE|Na symmetric cells achieved a cycle life of ∼4248 h at 0.5 mA cm^–2^ and 1 mAh cm^–2^, while Na|SPE|P2-type Na_2/3_[Ni_1/3_Mn_2/3_]­O_2_ composite cathode full cells displayed 80.6% capacity retention after 700 cycles at 1C, both of which are the highest reported values among SPE-based all-solid-state SMBs. This excellent performance was attributed to the combined mechanical and electrochemical properties of the SPE.

## Graphical abstract

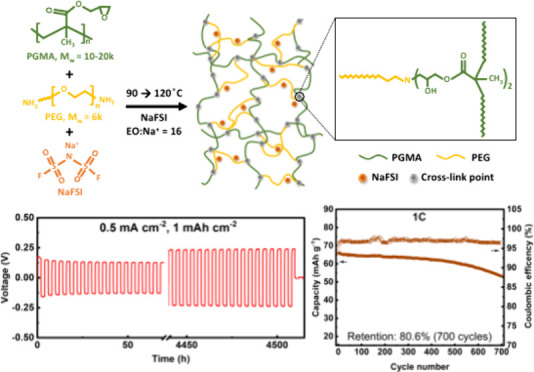

## 1 Introduction

Lithium-ion batteries have remained the standard for consumer electronics and electric vehicles. However, the high cost and unstable supply chains of the raw materials necessary for these batteries make them unsuitable for applications such as large-scale grid storage.^1,2^ As a result, sodium-metal batteries (SMBs) have recently gained increasing attention with comparable theoretical energy density (1166 mAh g^–1^ for Na vs 3860 mAh g^–1^ for Li) and much greater elemental abundance in the Earth’s crust (2.4 × 10^4^ ppm for Na vs 20 ppm for Li).^3,4^ Despite early success, SMBs using conventional liquid electrolytes suffer from numerous issues, including low Coulombic efficiency (CE), limited electrochemical stability window, and poor safety due to the high flammability of the electrolyte.^3,4^

One solution to address these challenges is employing solid electrolytes (SEs) to fabricate solid-state batteries (SSBs) because SEs offer several advantages, such as reduced reactivity, enhanced CE, broadened electrochemical stability window, increased safety, enhanced energy density, and reduced material cost.^5^ Much of the research efforts toward SE development have focused on inorganic-based materials due to their superior ionic conductivity. However, inorganic SEs are stiff and brittle, requiring more intensive processing compared to conventional bending and rolling techniques.^6^ The high stiffness of these materials also requires the use of either high stack pressures (≥1 MPa) or advanced interfacial engineering strategies such as alloying or atomic layer deposition to partially alleviate the large interfacial resistance at the electrodes.^7^

Solid polymer electrolytes (SPEs) offer inexpensive raw material cost and facile and scalable processing and can operate at lower stack pressure.^5,6,8^ SPE-based SSBs have already been commercialized by Blue Solutions, primarily in electric bus applications, thus motivating further development of this technology.^9^ Among the various molecular architectures studied, cross-linked elastomeric network SPEs have consistently displayed excellent cycling performance in alkali-metal batteries.^10–15^ This is attributed to their ability to maintain a compliant solid–solid interface and undergo minimal plastic deformation when subjected to the significant volume changes imparted by the metal electrode during battery charging and discharging.^8,14,16^ Cross-linked network SPEs can be synthesized by using photopolymerization of monomers with multiple acrylate chain ends,^17–19^ copolymerization of telechelic macromolecules,^11^ cross-linking via multifunctional cross-linkers, including inorganic nanoparticles such as polyhedral oligomeric silsesquioxane (POSS) or silica,^20–22^ and macromolecular comb-chains.^14^ Interpenetrating network SPEs have also been reported.^23–25^ Because SPEs are subjected to significant deformation during the charging and discharging of SSBs, it becomes crucial to tune the network structure to improve properties such as elasticity, toughness, and compliance, thereby accommodating the stresses associated with this deformation. Previous work has demonstrated the importance of SPE properties such as toughness and resilience (elastic toughness) in stabilizing lithium-metal battery deposition through the use of various molecular design strategies such as dynamic-covalent cross-linked networks,^26^ vulcanizate nitrile rubber networks,^27^ nanophase-separated networks,^28^ semi-interpenetrating networks,^29^ and polymer nanofiber composite electrolytes.^30^ Compared to lithium metal, sodium metal possesses higher reactivity due to its larger atomic radius than lithium (1.86 Å for Na vs 1.52 Å for Li),^31^ which facilitates easier electron donation to reduce the electrolyte. This, coupled with the lower bulk modulus of sodium (6.3 GPa for Na vs 11 GPa for Li),^31^ makes it more susceptible to orphaned metal formation, lowering the CE and battery capacity.^3,32^ Thus, designing SPEs with high toughness becomes essential to not only mitigate dendrite induced short circuit, but also reduce orphaned metal formation to improve cycling reversibility. While the previously mentioned studies have illustrated the impact of toughness in lithium metal battery performance, to our knowledge, no studies have established the effect of such properties in all-solid-state SMBs (ASSSMBs). Furthermore, the relationship between network structure and mechanical resilience, as well as the link between cyclic fatigue resistance and battery performance in ASSSMBs, has not yet been demonstrated in the literature.

In this work, we developed a novel molecular architecture for ASSSMBs that utilizes poly­(glycidyl methacrylate) (PGMA) as a comb-chain macromolecular cross-linker and amine-terminated poly­(ethylene glycol) (PEG) as the ion-solvating medium to form a polymer network.^14,33^ As shown in Scheme 1, epoxy groups distributed along each PGMA polymer chain provide numerous cross-linking points. The advantages of this high functionality cross-linker design are to accelerate gelation and improve long-range interconnectivity within the network.^14,34^ When fast gelation is promoted, phase separation during polymerization can be minimized, resulting in a homogeneous network.^34^ The combined effects of the homogeneous network structure and enhanced long-range interconnectivity of the network enable more uniform stress distribution, which leads to a toughness of 1.6 MJ m^–3^. The impact of SPE’s high mechanical toughness is reflected in its Na|SPE|Na symmetric performance, exhibiting the longest cycle life for reported sodium SPEs. The high toughness and flexibility of this SPE also make it an excellent choice as a catholyte material. P2-type Na_2/3_[Ni_1/3_Mn_2/3_]­O_2_ (NNMO), where “P” stands for Na-occupying prismatic sites and “2” represents the number of transition-metal layers in a repeating unit, was used as the active cathode material.^35^ When this SPE is utilized as a catholyte binder and separator, Na|SPE|NNMO-composite cathode full cells demonstrated excellent discharge capacities. Additionally, the high mechanical resilience of this design enabled highly reversible charging and discharging, displaying a capacity retention of 80.6% after 700 cycles at 1C, which, to our knowledge, is the highest capacity retention at this cycle number reported in the literature for polymer-based ASSSMBs.

**Scheme 1:**
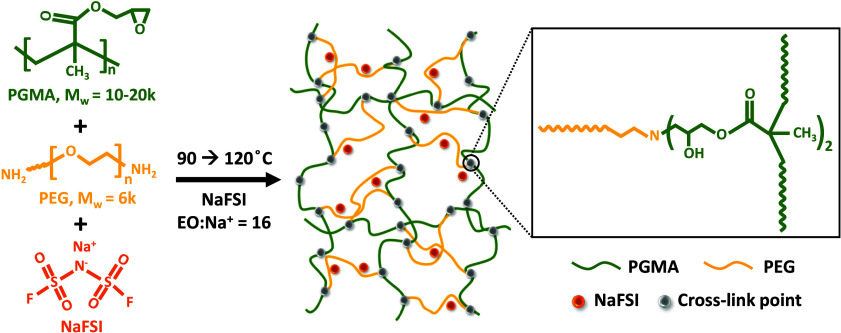
Synthetic Scheme of 4PGMA–PEG_6k_ SPE

## 2 Experimental Section

### 2.1 Materials

Poly­(glycidyl methacrylate) (PGMA; number-average molecular weight, *M*_n_ = 10–20 kDa), PEG diamine (PEG_6k_; *M*_n_ = 6 kDa), tetrahydrofuran (THF; ≥99.9%), dimethylformamide (DMF; 99.8%), and sodium metal (99.9% trace metals basis), sodium acetate trihydrate, nickel acetate tetrahydrate, manganese acetate tetrahydrate, and oxalic acid were purchased from Sigma-Aldrich. Sodium bis­(fluorosulfonyl)­imide (NaFSI; ≥98%) was purchased from TCI. Super P was purchased from MTI.

### 2.2 SPE Synthesis

PGMA, PEG_6k_, and NaFSI were dissolved in a mixed solvent consisting of 1/3 vol % DMF and 2/3 vol % THF at a total solid-to-solvent concentration of 200 mg mL^–1^. The solutions were cast onto a glass slide, and the samples were cured under vacuum at 90 °C for 24 h and 120 °C for an additional 12 h of postcuring. After curing, samples were cut into 12 mm × 12 mm films and then dried under vacuum for 12 h at 120 °C to remove moisture before transfer into an Ar-filled glovebox with <0.5 ppm of O_2_ and <0.5 ppm of H_2_O. The successful completion of the reaction was confirmed by Fourier transform infrared (FTIR) spectroscopy.

### 2.3 NNMO Synthesis and Composite Cathode Fabrication

NNMO (P2-type Na_2/3_[Ni_1/3_Mn_2/3_]­O_2_) was synthesized by solid-state conversion of Ni metal.^35^ In a typical synthesis process, 100 mg of metallic Ni powder (1 μm, 99.8%, Sigma-Aldrich) was mixed with 560 mg of sodium permanganate monohydrate (NaMnO_4_·H_2_O; 97%, Sigma-Aldrich) and 1 mL of a 1 M NaOH solution. The NaOH was used to provide an extra Na source to compensate for Na loss during high-temperature solid-state conversion. The mixture was heated on a hot plate at 150 °C in air to evaporate water, followed by ball-milling in an agate jar (Across International) for 1 h. Finally, solid-state conversion was performed at 800 °C for 24 h in a muffle furnace (Thermo Scientific) in air, followed by rapid cooling to room temperature.

NNMO composite cathodes were prepared by mixing active material, Super P, and 4PGMA–PEG_6k_ precursor with NaFSI (EO/Na 16:1) at the weight ratio 56:14:30 in DMF. The resulting slurry was coated onto aluminum foil using a doctor blade, then dried at 40 °C for 24 h under vacuum. After solvent removal, the cathode film was placed between two stainless steel plates and C-clamped together. The cathode was then cured at 120 °C for 12 h under vacuum. After curing, the cathode was calendared to 90–95% of its initial thickness by hot pressing at 90 °C for 30 min. Electrodes with mass loadings from 2.8 to 3.1 mg cm^–2^ were then punched and dried under vacuum for an additional 12 h at 120 °C to remove moisture before transfer into an Ar-filled glovebox with <0.5 ppm of O_2_ and <0.5 ppm of H_2_O.

### 2.4 Electrochemical Cell Preparation

All sodium-metal electrodes were prepared by slicing sodium-metal ingots and rolling them between two sheets of Mylar to a thickness of 600 μm. Sodium symmetric cells were prepared by sandwiching 85–90-μm-thick SPE films between two 8-mm-diameter sodium disks punched from the rolled foil. Coin cells were assembled using one 500 μm spacer and a washer spring and crimped under 1050 kg of pressure. Prior to cycling, the cells were annealed at 80 °C for 4 h and then precycled for six cycles at a current density of 0.04 mA cm^–2^ and an areal capacity of 0.12 mAh cm^–2^.

Na|4PGMA–PEG_6k_|NNMO–CC cells were prepared by sandwiching 15–20-μm-thick SPE films between 600-μm-thick, 8-mm-diameter sodium disks and 6-mm-diameter NNMO composite cathodes. Coin cells were then assembled using two 500 μm stainless steel spacers and a washer spring and crimped under 1050 kg of pressure. Prior to cycling, the cells were annealed at 80 °C for 4 h and then precycled for three cycles at a rate of 0.2C. For an active material mass loading of 2.8–3.1 mg cm^–2^, this corresponds to a current density of 0.048–0.054 mA cm^–2^.

### 2.5 Spectroscopy, Thermal, Mechanical, and Electrochemical Characterization

FTIR spectra were collected using a Bruker Invenio-R spectrometer with an ATR mode. The SPE films were sandwiched between the ATR crystal and the pressure arm throughout the scan. Scans were taken at 25 °C and a resolution of 2 cm^–1^, averaging 16 scans for background and sample collection.

Thermal measurements were conducted using a TA Instruments Q2000 differential scanning calorimeter, calibrated using indium. Samples were heated from 20 to 110 °C at 10 °C min^–1^, cooled to −90 °C at 10 °C min^–1^, and then heated again to 110 °C at 10 °C min^–1^. The heat–cool–heat thermal profile was used to understand the thermal history and cross-linking effect on the polymer thermal properties.

Thermogravimetric analysis (TGA) was performed on a TA Instruments Discovery TGA 5500. Samples were heated from 25 to 1000 °C at a scan rate of 10 °C min^–1^ under a nitrogen atmosphere.

Tensile measurements were conducted on a TA Instruments Discovery Hybrid Rheometer-3 (DHR-3) at 80 °C. Rectangular films of 15 mm × 5 mm × 0.15 mm were carefully tightened between the tensile clamps and then equilibrated at 80 °C for 2 min prior to measurements. Samples were drawn at a constant strain rate of 100% min^–1^. Strain cycling was also performed at a constant strain rate of 100% min^–1^ with 5 s rest periods between each cycle. Young’s modulus was determined by taking the slope of the stress–strain curve in the linear elastic regime of the SPE. Tensile strength was determined by taking the stress before material fracture. Elongation at break was taken as the strain before material fracture. Toughness was determined by integrating the area under the stress–strain curve. These values were averaged from three separate tensile experiments to determine the mechanical properties of the material.

Impedance measurements used to determine ionic conductivity were taken on a Parstat 2273 potentiostat. Films were sandwiched between two blocking stainless steel electrodes for all sample measurements. The samples were heated from 20 to 100 °C and annealed at 100 °C for 1 h. Impedance measurements were made on the first cooling cycle from 100 to 30 °C. Samples were held at each temperature for at least 15 min prior to measurements. Impedance measurements were taken over the frequency range of 0.1 Hz to 1 MHz with an applied potential of 60 mV. Bulk resistance values were determined by fitting the Randles circuit to the Nyquist plot to obtain the semicircle touchdown point. The resulting conductivities were calculated using the sample thickness *L* and the cross-sectional area *A*:

$$\sigma =\frac{L}{RA}$$

Thicknesses were determined using a micrometer after cell disassembly by cutting the films into four sections and averaging the measured thickness of each section.

Cyclic voltammetry (CV) was performed using a Gamry Interface potentiostat/galvanostatic/ZRA instrument using asymmetric Na|SPE|stainless steel cells. Scans were taken from 2 to 6 V at a rate of 0.1 mV s ^–1^.

### 2.6 X-ray Microcomputed Tomography and 3D Image Analysis

Laboratory-based microcomputed tomography (micro-CT) scans were performed on the samples using a Zeiss Xradia 620 Versa microscope (Carl Zeiss, Pleasanton, CA). Detailed data analysis can be found in the Supporting Information. In brief, two composite cathode samples were selected for X-ray tomography study: a pristine sample annealed at 80 °C for 4 h and a cycled sample tested at 1C and 60 °C for 300 cycles. First, the sodium anode was trimmed off with a sharp razor blade after the cell was disassembled. A 0.5 mm electrode disk was extracted from the samples using a biopsy punch and laid onto a sticky-tape-capped, 3-mm-diameter phenolic laminate rod (Figure S1 and Table S1). The assembly at the tip of the rod was encased in a 3-mm-inner-diameter polyimide capillary and sealed with a fast-setting epoxy. The sample mount preparation was performed in an Ar-filled glovebox with 0.1 ppm of O_2_ and <0.1 ppm of H_2_O. Afterward, the whole sample mount assembly was sealed in an aluminized pouch for transport to the micro-CT instrument and only opened when ready to scan.

## 3 Results and Discussion

### 3.1 Synthesis and Characterization of 4PGMA–PEG_6k_ SPE

Scheme 1 displays the synthetic route and molecular structure of the PGMA–PEG/NaFSI-based SPE. The SPE utilized in this work is denoted as 4PGMA–PEG_6k_, where 4 is the molar ratio of PGMA repeat units to PEG molecules and 6k is the PEG molecular weight. The network was formed by the reaction between the PGMA epoxide groups and amine end groups of the PEG, as shown in Scheme 1. Utilizing a PGMA repeat unit/PEG molar ratio of 4 ensures the stoichiometric ratio of epoxide and amine. The mass ratio of PGMA to PEG to NaFSI is 6.9:72.4:20.7. Because the average degree of polymerization of the PGMA cross-linker is 106, i.e., the functionality is *f* = 106, the critical branching coefficient is α_c_ = 1/(*f* – 1) ∼ 0.01. This suggests that gelation of the network would occur at an early stage of the reaction,^36^ leading to a uniform transparent film upon polymerization. The film is mechanically robust and can be easily handled by hand or tweezers (Figure S2). The FTIR spectra of PGMA, PEG_6k_, NaFSI, and the 4PGMA–PEG_6k_ SPE after curing are shown in Figure S3, which reveals that there is no epoxy peak corresponding to C–O–C asymmetric stretch in PGMA at 910 cm^–1^ after reaction, confirming the complete extent of reaction.

The thermal stability of the 4PGMA–PEG_6k_ SPE was determined using TGA (Figure S4). The 4GPMA-PEG_6k_ SPE displays <1 wt % weight loss until the first decomposition reaction is observed to begin at 275 °C. In the first decomposition reaction from ∼275 to 325 °C, a ∼37% loss in mass is observed, which is most likely attributed to the decomposition of the NaFSI salt.^37^ The second decomposition reaction from ∼326 to 440 °C shows an additional mass loss of ∼46%, likely due to the combination of polymer degradation with continuous decomposition of the salt. TGA results confirmed that the SPE is safe to use at a temperature as high as 275 °C. The thermal transitions of the 4GPMA–PEG_6k_ SPE were determined using differential scanning calorimetry (DSC). Figure 1a displays the second heating profile of the 4GPMA–PEG_6k_ SPE, 4GPMA–PEG_6k_-NNMO-based composite cathode (NNMO–CC), and salt-free monomers, while the thermal properties are tabulated in Table S2.

**Figure 1:**
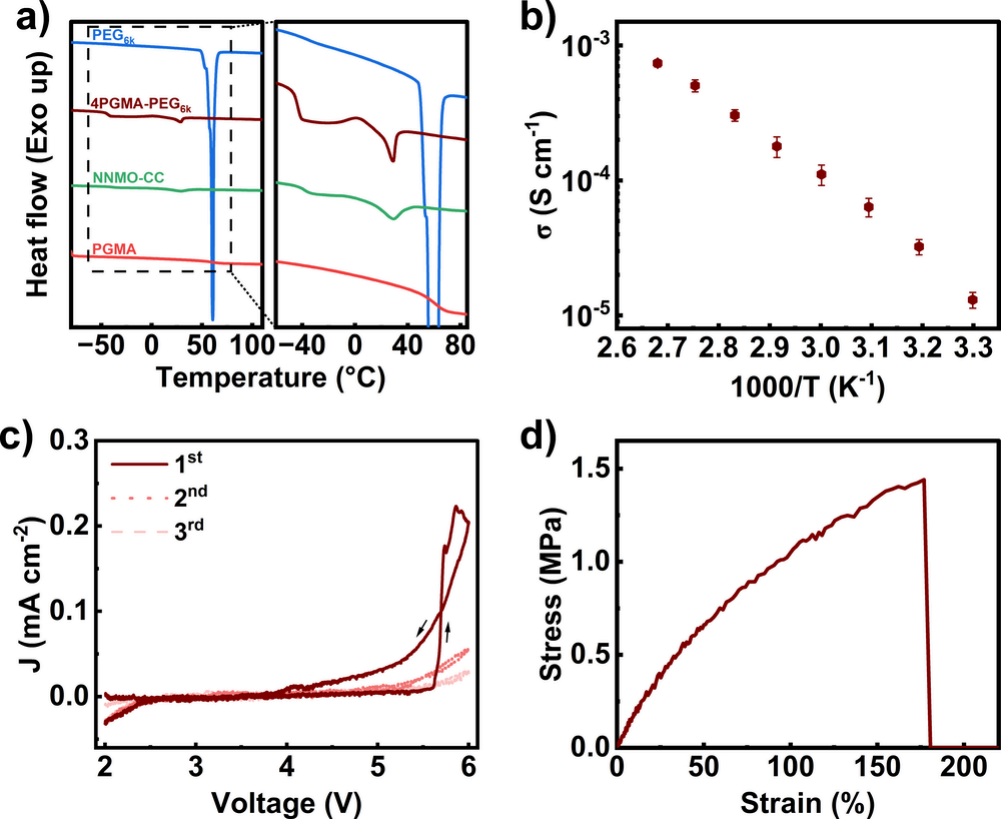
(a) DSC second heating thermograms of the 4PGMA–PEG_6k_ SPE, NNMO–CC, and salt-free monomers. (b) Temperature-dependent ionic conductivity of 4PGMA–PEG_6k_. (c) Cyclic voltammogram of Na|4PGMA–PEG_6k_|stainless-steel cell from 2 to 6 V at 80 °C. (d) Representative stress–strain curve of 4PGMA–PEG_6k_ at 80 °C.

The DSC thermograms show a major melting peak for PEG_6k_ at 60.9 °C, confirming the highly crystalline nature of PEO. The typical step-transition of the glass transition is unclear from the figure due to the high crystallinity of PEG_6k_ and less amorphous PEO contributing to the transition in the second heating. PGMA shows a glass transition temperature of 62 °C. The 4PGMA–PEG_6k_ SPE displays a clear glass transition at −44.1 °C, a recrystallization peak at ∼0 °C, and a melting peak with a significantly lower *T*_m_ at ∼29.0 °C. The net heat of fusion (melting enthalpy minus the recrystallization enthalpy) is ∼3.0 J g^–1^, suggesting a very low *X*_c,PEO_ of 1.5%. This indicates that crystallization is mostly suppressed in the SPE, consistent with the observation of the glass transition in the DSC scan. The low *X*_c,PEO_ of the SPE can be attributed to the formation of the network structure and the inclusion of salt, both of which significantly hinder the chain rearrangement needed for polymer crystallization. The NNMO–CC, however, displays a higher *T*_g_ of −38.8 °C, a higher *X*_c,PEO_ of 10.0%, and a higher *T*_m_ of ∼29.3 °C when compared to the neat 4PGMA–PEG_6k_ SPE. The DSC experiments therefore confirm that (1) PEG crystallinity is significantly suppressed at room temperature for both the SPE and the NNMO–CC, (2) there are no PEG crystals above ∼40 °C for both the SPE and the NNMO–CC, (3) the higher crystallinity of NNMO–CC compared to neat SPE is likely due to the potential disruption of the cross-linking network by the NNMO particles, and (4) the higher *T*_g_ of the NNMO–CC is ascribed to retardation of the segmental mobility imparted by the NNMO particles, a phenomenon commonly observed in polymer composites.^38,39^

Figure 1b presents the temperature-dependent ionic conductivity of the 4PGMA–PEG_6k_ SPE, which displayed a conductivity of 1.3 × 10^–5^ S cm^–1^ at 30 °C, 1.1 × 10^–4^ S cm^–1^ at 60 °C, and 3.0 × 10^–4^ S cm^–1^ at 80 °C. These values are consistent with our previous work.^14^ The higher conductivity achieved by the 4PGMA–PEG_6k_ SPE compared to linear PEO-based SPEs at 30 °C (<10^–6^ S cm^–1^) can be attributed to the efficient cross-linking reaction, which largely suppresses crystallization (*X*_c,PEO_ = 1.5%), and the relatively long PEG chain length (6k), which enhances segmental mobility. Ionic conductivity in polymer electrolytes is known to follow VTF temperature dependence:^40,41^

1 $$\sigma =AT^{-1/2}\;\exp\left[\frac{E_{a}}{R\left(T-T_{0}\right)}\right]$$

where the prefactor *A* is related to the number of charge carriers, *E*_a_ is related to the activation energy of ion transport, and *T*_0_ is the Vogel temperature (*T*_g_ – 50). The conductivity was fitted to the VTF equation (Figure S5) to obtain *A* and *E*_a_ and values of 14.6 S cm^–1^ K^1/2^ and 11.3 kJ mol^–1^, respectively, which is in good agreement with linear PEO.^42^

The electrochemical stability was evaluated by CV of a Na|4PGMA–PEG_6k_|stainless-steel cell, scanning between 2 and 6 V (Figure 1c). A scan rate of 0.1 mV s^–1^ was chosen because it is on the order of a charge/discharge rate of 0.1C. An oxidative stability of 5.6 V vs Na/Na^+^ was determined by extrapolating tangent lines between the baseline current prior to oxidation and the first current peak, following the method described in a previous work.^43,44^ It remains unclear whether stainless steel, SPE, or both contribute to the onset of oxidative current; however, the SPE stability limit is determined to be at least 5.6 V vs Na/Na^+^. The high oxidative stability of the 4PGMA–PEG_6k_ is likely due to the rich ester functionality from PGMA, which is consistent with our previous work.^14^ It therefore ensures its compatibility with high potential electrode materials.

Another interesting feature of the CV results is that the magnitude of the maximum current density decreased by ∼75% from the first to second scan and ∼50% from the second to third scan, suggesting that a passivation layer forms on the stainless-steel electrode. Liquid electrolytes containing FSI anions are commonly observed to show the opposite trend, where increases in the current on ensuing CV scans indicate that electrode corrosion becomes more severe with increased cycling. The oxidation of stainless steel in electrolytes containing FSI anions is believed to proceed through a mechanism similar to that in aluminum, where polarization leads to the formation of Al^3+^, which will subsequently form Al­(FSI)_3_ complexes with anions.^45,46^ Dissolution of this layer is promoted by low-concentration electrolytes, where there is a higher quantity of free solvent and anion molecules,^47^ as well as by electrolytes with a high dielectric constant.^48^ Thus, the passivating effect exhibited by the 4PGMA–PEG_6k_ SPE is likely 2-fold: (1) lower solubility of the reduction products in the ether-based SPE and (2) retarded diffusion kinetics in the SPE, preventing dissolution and transport of the oxidation products from the electrode surface.

Elasticity and toughness have been shown to be critical SPE properties for both maintaining a compliant electrode interface and enduring the repeated stresses experienced in alkali-metal batteries with minimal plastic deformation.^14,26,29,49,50^ Figure 1d displays the representative stress–strain curve for the 4PGMA–PEG_6k_ SPE, while the mechanical properties averaged from three tensile experiments are shown in Table S3. The 4PGMA–PEG_6k_ SPE shows high toughness and elongation at break of 1.6 MJ m^–3^ and 181%, respectively. These excellent mechanical properties are superior to previously reported small-molecule or nanoparticle-cross-linked SPE networks, which displayed toughness ≤ 0.5 MJ m^–3^.^19,29,51^ The observed high toughness is attributed to the comb-chain cross-linker. As previously discussed, the PGMA cross-linker ensures an extremely low critical branching coefficient of ∼0.01, which leads to fast network formation kinetics. This would produce a homogeneous network topology, ensuring more uniform stress distribution across the elastically active network strands, thereby enabling greater elongation before failure. To better understand the fatigue resistance of the 4PGMA–PEG_6k_ SPE, the SPE was repeatedly strain-cycled to 50% strain (Figure S6). The first cycle shows a small hysteresis loop, which corresponds to a dissipated energy of ∼21% of the initial 0.12 MJ m^–3^ under the loading curve. The small hysteresis loop upon unloading in the stress–strain curve indicates primarily elastoviscous deformation,^52^ with the dissipated energy loss attributed to chain scissoring of shorter network strands. By comparison, linear PEO exhibits a much greater dissipated energy of ∼70% after the first cycle in compressive stress tests up to only 10% strain.^53^ Upon subsequent cycles, there is virtually no hysteresis in our sample, confirming the deformation is elastic and recoverable. Additionally, the 4PGMA–PEG_6k_ SPE shows minimal strain softening from the 1st to 60th cycle, with a reduction in elastic modulus from 1.4 to 1.2 MPa (∼14%).

### 3.2 Symmetrical Cell Stripping/Plating Tests

To evaluate the resistance to dendrite propagation in the 4PGMA–PEG_6k_ SPE, galvanostatic stripping/plating experiments were employed in Na/Na symmetric cells. Two symmetric cells were cycled at a current density of 0.5 mA cm^–2^ and a capacity of 1 mAh cm^–2^. The average life obtained was an impressive 4248 ± 261 h. Figure 2b compares the cycle life with other SPEs and solid composite electrolytes (SCEs) reported in the literature. To the best of our knowledge, this represents the longest cycling life reported at the relatively high current density and capacity, as depicted in Figure 2b and Table S4. All of the previously reported literature in Figure 2b utilize linear SPEs or SCEs, with the exception of the POSS–PEG system. The markedly improved cycle life in our work, therefore, likely arises from differences in molecular architecture. If we consider 1 mAh cm^–2^ of sodium plating to result in a theoretical layer thickness of 8.8 μm, this corresponds to a compressive strain on the SPE of ∼10%. As previously discussed, linear PEO displays ∼70% dissipated energy in compressive stress tests up to 10% strain, indicating considerable plastic deformation of the polymer. Conversely, the 4GPMA–PEG_6k_ SPE displays nearly linear stress–strain behavior up to 10% strain and only 21% dissipated energy up to 50% strain. Thus, in linear SPEs, dendrite-induced stress likely leads to significant chain disentanglement, creep, and permanent deformation, which promotes faster dendrite propagation. In the case of the 4PGMA–PEG_6k_ SPE, the excellent mechanical resilience of the comb-chain cross-linker-based network structure accommodates large deformation induced by a growing sodium protrusion with minimal plastic deformation of the SPE. During the initial stages of cycling, the overpotential gradually decreases over the first 30 h until it stabilizes at ±130 mV, likely due to the formation of a more stable SEI layer. The voltage profile exhibits a square-wave shape that tracks the current input and lacks the sharp peaks associated with mossy dendrite formation and electrode pitting that are observed in liquid electrolyte systems.^54,55^ The absence of these voltage extrema using the 4PGMA–PEG_6k_ SPE indicates that the formation of such deleterious morphology is largely mitigated, which explains how such a long cycling life (∼6 months) was achieved.

**Figure 2:**
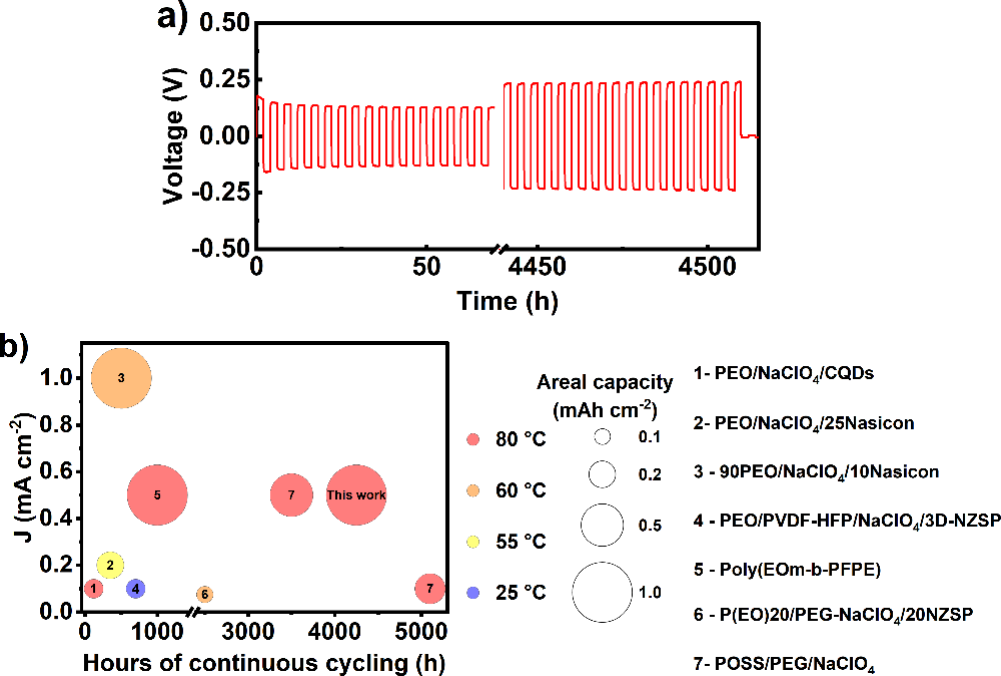
(a) Symmetric cell cycling for the 4PGMA–PEG_6k_ SPE at a current density of 0.5 mA cm^–2^ at 80 °C. (b) Current density versus hours of cycling plot for leading all-solid-state SPE/SCEs reported in the literature. The size and color of the symbols correspond to the areal capacity and temperature of the tests.

To better understand the underlying degradation mechanism of the sodium symmetric cells, cross-sectional scanning electron microscopy (SEM) images and the associated EDS elemental maps (Na, O, and C) were obtained for both the as-prepared annealed cell and the cycled cell after short circuit, as shown in Figure 3.

**Figure 3:**
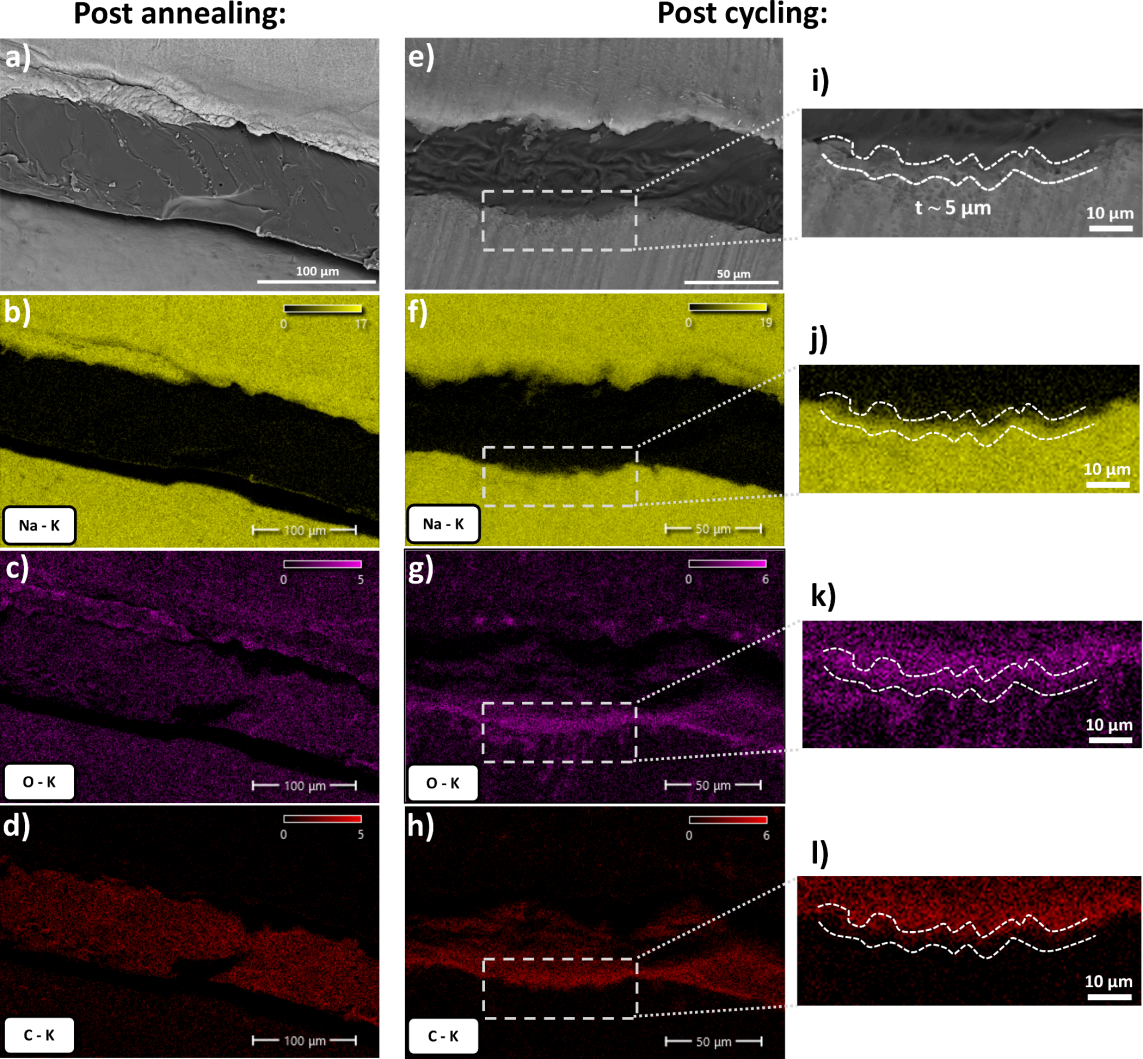
Cross SEM and EDS of symmetric cell cross sections: (a–d) after annealing and (e–l) after short circuit at 4510 h at a current density of 0.5 mA cm^–2^ and a capacity of 1 mAh cm^–2^.

The initial uncycled sodium cell displays a thickness of ∼85 μm and a gap between the bottom electrode and SPE, which is attributed to the sectioning process. After cycling, the SPE displays a reduction in thickness of ∼40% to ∼60 μm with no observable dendrites at the interface. The EDS maps of Na, O, and C reveal a discrete interface between the sodium metal and SPE, characterized by a thin interlayer (∼5 μm), where overlapping signals of Na, O, and C are observed, indicating the formation of a Na-SPE composite SEI layer outlined by the white dashed lines in Figure 3i–l). Migration of the sodium-metal electrode interface into the SPE during cycling has previously been observed in a POSS–PEG-based network system.^13^ The POSS–PEG SPE displays a reduction in SPE thickness of ∼80% after 3550 h of cycling at a current density of 0.5 mA cm^–2^ and a capacity of 0.5 mAh cm^–2^, with large spherulite-like dendrites suspended in the SPE and a much less clearly distinguishable interface between the SPE and sodium electrode.^13^ The significantly improved cycling performance observed in this work is likely due to the combined effects of an enhanced polymer network structure and the use of NaFSI salt because NaFSI has been shown to lead to the formation of a Na–F rich solid electrolyte interphase (SEI), which promotes more uniform deposition.^37^ Figure S7 shows the C 1s and F 1s X-ray photoelectron spectroscopy (XPS) spectra of the sodium symmetric cell anode in the pristine state and after sputtering with a 2 kV Ar ion gun for 1, 3, and 5 min. The XPS spectra reveal ∼1.8× and 1.5× increases in the intensity of the C–O and C–C/C–H peaks attributed to the polymer SEI degradation products, while the Na–F peak displays a 2.5× increase in magnitude after 1 min of sputtering. Further sputtering up to 5 min shows a marginal reduction of the intensity of the C–O and C–C/C–H peaks and an increase in the intensity of the Na–F peak. This suggests that the inner layer of the SEI is rich in stable Na–F, which helps protect the SPE from further reduction into the SEI. Thus, the above results suggest that the superior resilience of the SPE effectively mitigates dendrite-induced plastic deformation of the network that would otherwise promote orphaned metal formation during cycling, while the Na–F-rich inner SEI layer provides an electrochemically stable boundary which minimizes further reduction of the SPE into the SEI. This underscores the critical role of mechanical resilience and SEI electrochemical stability in SPE design for SMBs because dendrite propagation can lead to substantial electrolyte consumption and ultimately result in cell failure.

### 3.3 Full Cell Demonstration in Na|SPE|NNMO–CC Batteries

Another major advantage of SPEs with high toughness is that they allow for the fabrication of ultrathin SPE membranes (15–20 μm). Minimizing the SPE thickness is advantageous for overcoming limitations in ionic transport and maximizing energy density and power output.^56^ To this end, Na|SPE|NNMO–CC full cells with a 2.8–3.1 mg cm^–2^ NNMO active material mass loading were prepared to evaluate their electrochemical performance. Parts a and b of Figure 4 shows the rate performance of the corresponding Na|SPE|NNMO–CC full cells at 60 and 80 °C. At 80 °C, the cell displayed reversible capacities of 80, 77, 72, 44, 28, 19, and 13 mAh g^–1^ at 0.4, 1, 2, 4, 6, 8 and 10C, respectively. When cycling at 0.4C again, the cell achieved a capacity of 78 mAh g^–1^, which is very close to the theoretical capacity of NNMO at 86 mAh g^–1^. These are the highest capacity utilizations reported in the literature for pure SPE-based NNMO ASSSMBs at these current densities and active material mass loadings; comparable systems are listed in Table S5. The galvanostatic charge/discharge curves shown in Figure 4c reveal relatively flat plateaus for C rates of less than 2C, indicating the storage process is Faradaic in the ASSSMBs.

**Figure 4:**
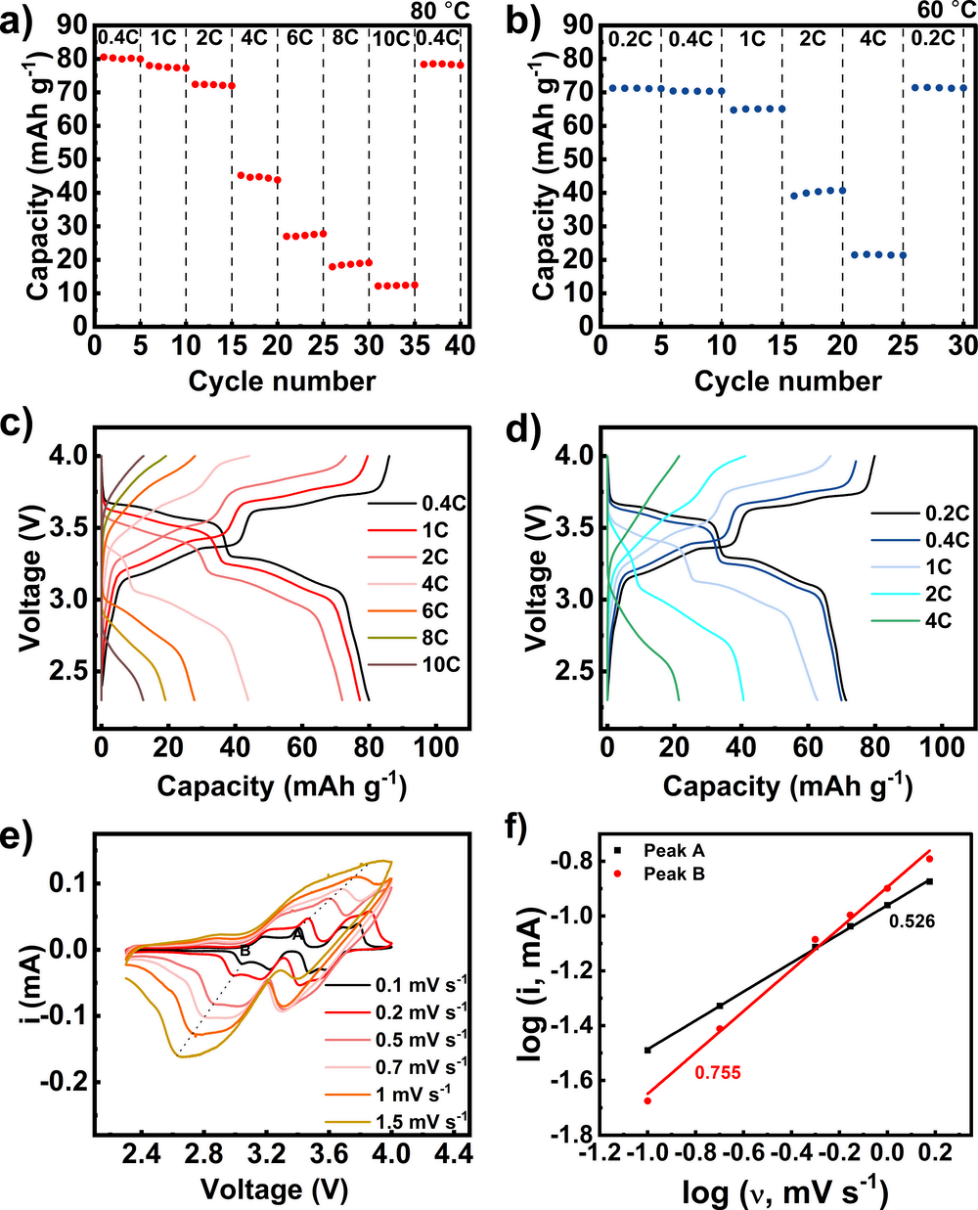
Discharge capacity versus cycle number and voltage profiles for rate capability experiment of Li|4PGMA–PEG_6k_|NNMO–CC cells at (a and c) 80 °C and (b and d) 60 °C. (e) CV curves of Na|4PGMA–PEG_6k_|NNMO–CC cells at different scan rates. (f) Linear relationship between log peak current and log scan rate, highlighting the calculated *b* values.

Micro-CT was performed on the NNMO–CC after crimping and annealing of a Na|4PGMA–PEG_6k_|NNMO–CC cell at 80 °C for 4 h in order to understand the intrinsic porosity and pore distribution within the cathode prior to cycling. Parts a and b of Figure S8 show the pore distribution within the 3D reconstruction of the composite cathode, while Figure S8c displays the pore-size distribution. The NNMO–CC displays an overall porosity of 1.6%, consistent with reported work.^57^ It is important to note that minimizing porosity in the cathode of SSBs is crucial for reducing ion transport tortuosity and enhancing capacity utilization.^58,59^ The high rate capability of the cells mentioned previously is thus attributed to the combined effects of the thin (25–30 μm), dense (1.6% porosity) composite cathode and the ultrathin (15–20 μm) SPE separator.

When the temperature is reduced to 60 °C, there is a drop in specific capacity, but the cell is still able to exhibit reversible capacities of 71, 70, 65, 41, and 21 mAh g^–1^ at 0.2, 0.4, 1, 2, and 4C, respectively (Figure 4b). When the rate was switched back to 0.2C, the cell delivered a high reversible capacity of 71 mAh g^–1^. The lower capacity utilization at 60 °C compared to that at 80 °C can be attributed to larger resistances within the cell as shown by EIS measurements taken after precycling at 80 °C and cooling to 60 °C (Figure S9) and as evidenced by the increased overvoltage in the potential profiles for 60 °C (Figure 4d) compared to 80 °C (Figure 4c). The bulk electrolyte resistance is indicated by the high-frequency intercept of the EIS trace. The semicircle at a lower frequency shows the Na/electrolyte interfacial and charge transfer resistance, while the semicircle at the lowest frequency represents the cathode/electrolyte interfacial resistance.^60,61^ The bulk resistance shows a very small contribution to the overall resistance and only increases from ∼8 to ∼20 Ω cm^2^ when lowering the temperature from 80 to 60 °C. The combined anode and cathode electrolyte interfacial resistances, however, display a much stronger temperature dependence because they increase by ∼3.2× from ∼600 to 2000 Ω cm^2^, which indicates that these resistances are the main rate-limiting factors.

To better understand the kinetics of sodiation/desodiation in the NNMO composite cathode, CV scans were taken at various rates of 0.1–1.5 mV s^–1^ between 2.3 and 4.0 V (Figure 4e). Two sets of redox peaks are seen, which are attributed to the reversible sodiation and desodiation of 1/6 Na from NNMO and Ni^2+^/Ni^3+^ redox. The contribution of the capacitive behavior that arises from a surface-controlled process can be estimated by extrapolating the redox peak current (*i*) at different scan rates (ν).^62^ The degree of capacitive contribution can be obtained from the relationship

2 $$i=a\nu^{b}$$

where *a* and *b* are variable parameters. When *b* is close to 0.5, the reaction is dominated by Faradaic intercalation (diffusion limited), and when *b* gets closer to 1, the process is more capacitive.^63,64^ Figure 4f shows the log­(*i*)–log­(ν) relationship attained from log­(*i*) = log­(*a*) + *b* log­(ν), where the cathodic and anodic peak currents are used. The cell is scanned at different sweep rates ranging from 0.1 to 1.5 mV s^–1^, and the values of *b* for both anodic (A) and cathodic (B) peaks are 0.526 and 0.755, respectively. These values match well with NNMO scanned in liquid electrolyte (0.58 for A and 0.63 for B)^65^ and indicate fast charge-transfer kinetics, particularly for an all-solid-state battery, which is consistent with the high rate capability exhibited by the cell in the previous section.

The long-term cycling stability of the 4PGMA–PEG_6k_-based SSBs was evaluated in full cells that were cycled at rates of 1C and 2C at 60 and 80 °C, as shown in Figure 5. At mass loadings of 2.8–3.1 mg cm^–2^, these rates correspond to current densities of 0.24–0.26 and 0.48–0.52 mA cm^–2^ for 1C and 2C, respectively. Over 700 cycles at a rate of 1C at 80 °C, the cell maintains a capacity retention of 80.6%, corresponding to a capacity loss of just 0.028% per cycle. This performance ranks among the best reported in the literature for this rate and cycle number, as shown in Table S5. During the first 400 cycles, the cell displays a relatively constant capacity loss rate of 0.015% per cycle with a retention of 94.2%. After this point, the capacity retention begins to deteriorate at an increasing rate, and the cell exhibits a loss of 0.045% per cycle in the last 300 cycles. This nonlinear aging behavior is commonly observed in the later stages of battery cycling and is typically attributed to severe deterioration of ion transport kinetics.^66,67^ Figure 5d shows a more dramatic increase in overvoltage from the 500th cycle to the 600th and 700th cycles, suggesting greater impedance growth in the cell. This is likely because the gradual reduction of the SPE into the sodium SEI layer results in the formation of a thick, resistive layer that hinders ionic transport. This issue becomes exacerbated with continued cycling, leading to the observed nonlinear capacity decay.

**Figure 5:**
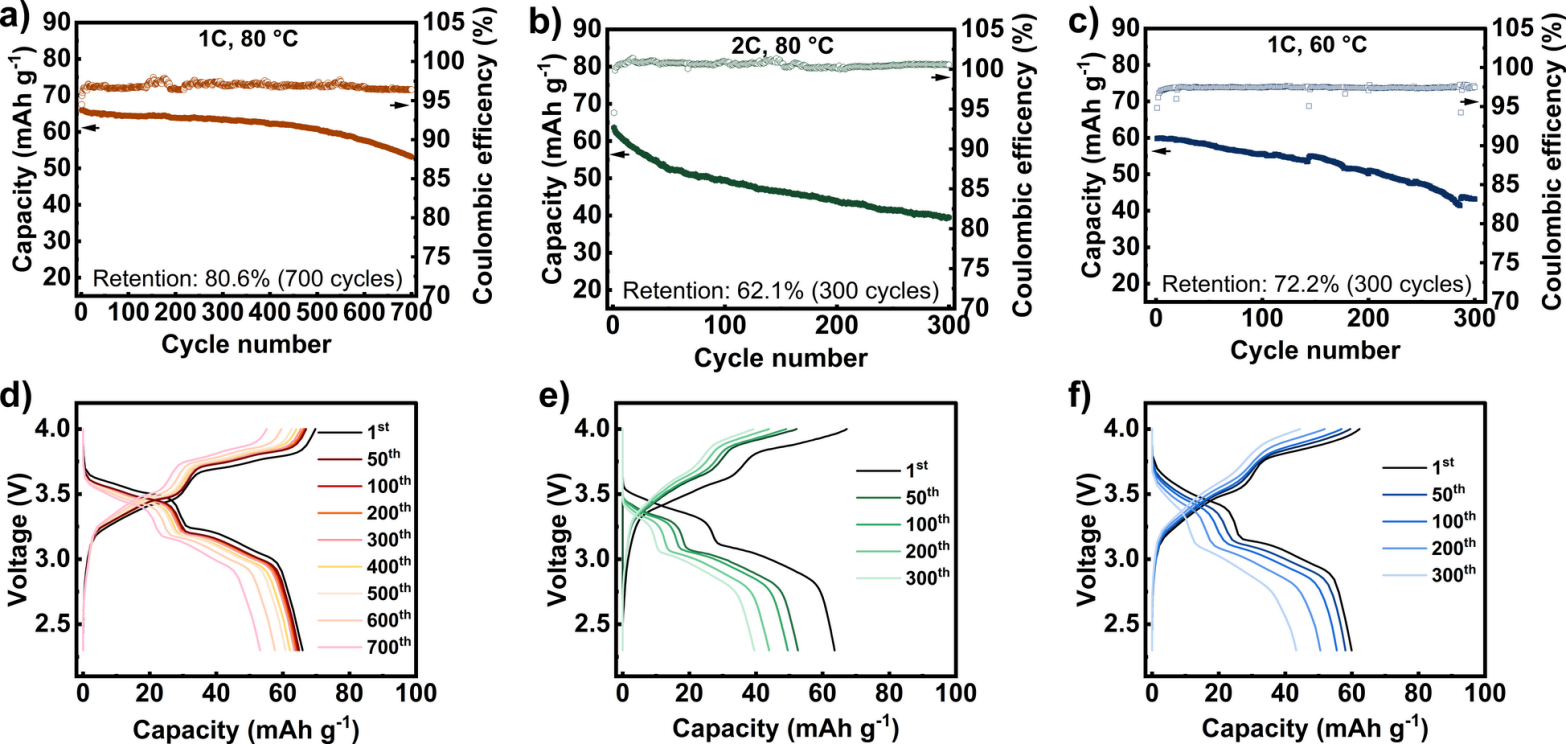
Discharge capacity/CE versus cycle number and charge/discharge profiles at (a and d) 1C, 60 °C, (b and e) 1C, 80 °C, and (c and f) 2C, 80 °C.

At a rate of 2C at 80 °C, the cell exhibits a capacity retention of 62.1% after 300 cycles (Figure 5b). During the first 50 cycles, the cell displays a faster capacity decay of 0.36% per cycle with a retention of 82.3%. After 50 cycles, the degradation rate begins to slow to a rate of 0.08% for 250 cycles. The more rapid capacity deterioration in the first 50 cycles was also observed in a second cell run under the same conditions (Figure S10). Slowdown of the capacity degradation rate under fast charging conditions has been observed previously in full cells with layered oxide-based cathode materials. It was attributed to the reduction of electrolyte SEI formation on the Ni_0.80_Co_0.15_Al_0.05_O_2_ cathode.^68^ Once a robust SEI was formed, the rate of capacity loss effectively slowed. It is possible that in our case, the use of a higher rate provides greater activation energy for such SEI decomposition reactions to occur. After a sufficiently thick SEI layer is formed, the reaction slows along with the capacity degradation rate.

When cycled at a rate of 1C at 60 °C, the cell exhibits a capacity retention of 72.2% after 300 cycles (Figure 5c). Previous work has demonstrated that inorganic electrolytes used as catholyte materials can suffer from severe capacity degradation when paired with active material undergoing volume changes of 4% or larger.^69,70^ This is caused by contact loss between the active material and the catholyte. To better understand the influence of catholyte mechanical properties on its ability to accommodate volume change of the active material, micro-CT of the NNMO–CC after cycling was taken, showing the pore distribution (Figure S11a,b) and pore size as a function of depth into the cathode (Figure S11c). After cycling, the NNMO–CC shows a lower concentration of pores near the interface, a reduced pore density and increased average pore size compared to the pristine state (Figures S8 and S11 and Table S6). Additionally, the overall porosity decreases from 1.6% before cycling to 0.5% after, suggesting cycling reduces porosity in the NNMO–CC. The reduced pore density at the interface could be due to plastic deformation and creep of the SPE around the NNMO–CC, which would result in a more conformal interface and may explain the small increase in capacity in the early stages of cycling. Thus, the 4PGMA–PEG_6k_ catholyte binder demonstrates the ability to successfully accommodate the volume change of the active material during cycling without growth of the total void volume. Previous work by Shi et al. showed that solid-state cells with a LiNi_0.5_Mn_0.3_Co_0.2_O_2_|Li_2_O–ZrO_2_(LZO)|carbon-based composite cathode exhibited over 85% capacity degradation after 50 cycles at 0.05 mA cm^–2^.^69^ They attributed this degradation to contact loss between the LZO catholyte and NMC active material, as revealed by FIB-SEM 3D reconstruction, which showed an increase in the void volume from 3% to 9.5% before and after cycling. This contact loss was linked to plastic deformation and cracking of the LZO catholyte, given the limited elastic strain of LZO. The reason the NNMO–CC does not exhibit porosity growth with cycling when compared to LZO is most likely due to the excellent ductility of the 4PGMA–PEG_6k_ SPE (∼180% strain at break). High ductility would enable the catholyte binder endure the strain induced by sodiation/desodiation of the NNMO active material without crack formation or fracture, highlighting the importance of this material property in effective catholyte design.

## 4 Conclusion

In this work, we designed a novel comb-chain cross-linked SPE for ASSSMBs. The fast gelation kinetics employed by utilizing a high-functionality PGMA macromolecular cross-linker were shown to effectively suppress crystallization and phase separation, leading to a more uniform network structure and enhanced mechanical properties. This resulted in high conductivity at 30 °C of 1.3 × 10^–5^ S cm^–1^, while maintaining an excellent elongation at break of 181% and toughness of 1.6 MJ m^–3^. Improving these mechanical properties enhanced sodium dendrite resistance in Na|SPE|Na symmetric cells, where a cycle life of 4248 h was achieved at a current density of 0.5 mA cm^–2^ and a capacity of 1 mAh cm^–^^2^. Implementation of this SPE design into Na|SPE|NNMO-composite cathode full cells showed a capacity retention of 80.6% after 700 cycles at 1C. These results represent the longest symmetric and full-cell cycle lifetimes reported in the literature for SPE-based ASSSMBs. Our results demonstrate the critical role of toughness and resilience in the suppression of dendrite propagation and orphaned metal formation to enhance the cycling efficiency in ASSSMBs.

## Supplementary Material



## Notes

X-ray microtomography analysis, digital photographs of SPE films, FTIR, TGA, VTF fitting of conductivity, strain cycling tests, SEM images of 4GPMA–PEG_6k_-CC, and EIS characterization (PDF)
