# Supplementary material for: A Comb-Chain Cross-Linker-Based Network Solid Polymer Electrolyte for All-Solid-State Sodium-Metal Batteries
Source: ACS Appl Energy Mater. 2025 Sep 11;8(18):13959–69. doi: 10.1021/acsaem.5c02367 (PMC12458455; doi:10.1021/acsaem.5c02367)
Supplement: Supplementary file 1 [file ae5c02367_si_001.pdf]

Supporting information

A Comb-Chain Crosslinker-Based Network Solid Polymer Electrolyte for All-Solid-State Sodium-Metal Batteries

*William R. Fullerton[1], Haoruo Liu[2], David N. Agyeman-Budu[3], Jintao Fu[2], Mohamed H. Hassan[2], Mark C. Staub[4], Eric Detsi[2], Johanna Nelson Weker[3] and Christopher Y. Li[1]**

[1]Department of Materials Science and Engineering, Drexel University, Philadelphia, PA 19104, USA

[2]Department of Materials Science and Engineering, University of Pennsylvania, Philadelphia, PA, 19104, USA

[3]Stanford Synchrotron Radiation Lightsource, SLAC National Accelerator Laboratory, Menlo Park, CA, 94025, USA

[4]TA Instruments, New Castle, DE, 19720, USA

*corresponding author e-mail: chrisli@drexel.edu

X-ray micro-computed tomography and 3D image analysis

Lab based micro-CT scans were performed on the samples using the Zeiss Xradia 620 Versa microscope (Carl Zeiss, Pleasanton CA, USA). A side view picture of the mounted sample and measurement layout are shown in **Figure S1**. The setup comprises of a broadband bremsstrahlung tungsten tube source that was operated at low voltage and power settings of 40 kV and 3.0 W to obtain a sufficient X-ray transmission (~20%) and image contrast in commensurate with the low density NNMO/polymer composite cathode (NNMO-CC) without using a filter. The detection was a 2048 × 2048 pixel array CCD detector coupled with a 20× objective lens. The data acquisition settings were adjusted for each sample to optimize the signal-to-noise level, namely, > 5 K raw counts per second. Thus, the parameters used for the sample-to-source distance, sample-to-detector distance, exposure time, and image pixel bin size settings for both the pristine NNMO-CC and the NNMO-CC after 300 cycles at 60 °C are given in **Table S1**. These settings also resulted in pixel sizes of 0.49 μm and 1.04 μm for the pristine and cycled samples, respectively. 1601 radiographs were collected for each scan over a 360° sample rotation. The images were automatically reference-corrected and reconstructed after determining the center shift and beam hardening parameters in using Zeiss Sout-and-Scan™ Control System Reconstructor (version. 16.2.18058.47373) software package.

The reconstructed 3D volume slices were imported into Dragonfly 2021.1 (Comet Technologies Canada Inc.), an advanced platform for scientific image processing for subsequent processing and segmentation to analyze the pore structure of the NNMO-CC. On importing into Dragonfly, the pixel size of the control was down sampled from 0.49 μm to match that of the NNMO-CC after 300 cycles at 60 °C dataset at 1.04 μm. Prior to segmentation, a volume of 220 × 90 × 25 μm^3 was cropped out for analysis and preprocessed by applying a total variation

smoothing filter[1] (Tv_chambolle) followed by an unsharp filter. To separate the SPE, void space (labeled as pores), and the composite NNMO/polymer volumes, we trained and applied a deep learning model U-net convolutional neural network for semantic segmentation of the features.[2] By using the segmentation wizard module within Dragonfly, we iteratively labeled, trained, and evaluated up to 8 represent frames to classify the images while monitoring the score of the model. After getting satisfactory training results and scores (a target of 0.99+), the trained model was applied to segment the rest of the dataset into regions of interest (ROI).

Next, we performed morphological and Boolean operations on the ROIs to further refine the pore classification in the samples. For that, we classified the segmented pores into three categories – outside, surface, and interior pores. The outside pores are in the SPE and located outside the boundary of the NNMO-CC ROI. The surface-connected pores are within the NNMO-CC ROI and are in direct contact with the SPE/NNMO interface at the boundary. The interior pores have no connection to the SPE/NNMO interface and are buried within the NNMO-CC. A post segmentation cleanup was performed on the pore ROI to remove noise by eliminating pores with cubed voxel count less than 9 (i.e. 2×2×2 voxels with volume < 9 μm^3). To spatially track the segmented pores as a function of depth from the SPE/NNMO interface, a Euclidean distance transform is applied from the SPE at the SPE/NNMO interface in a direction into the NNMO-CC. A distance grayscale map in 3D space is encoded and applied to both surface and interior pores such that the statistical analysis of the pore structure mapped as a function of depth into the NNMO-CC.

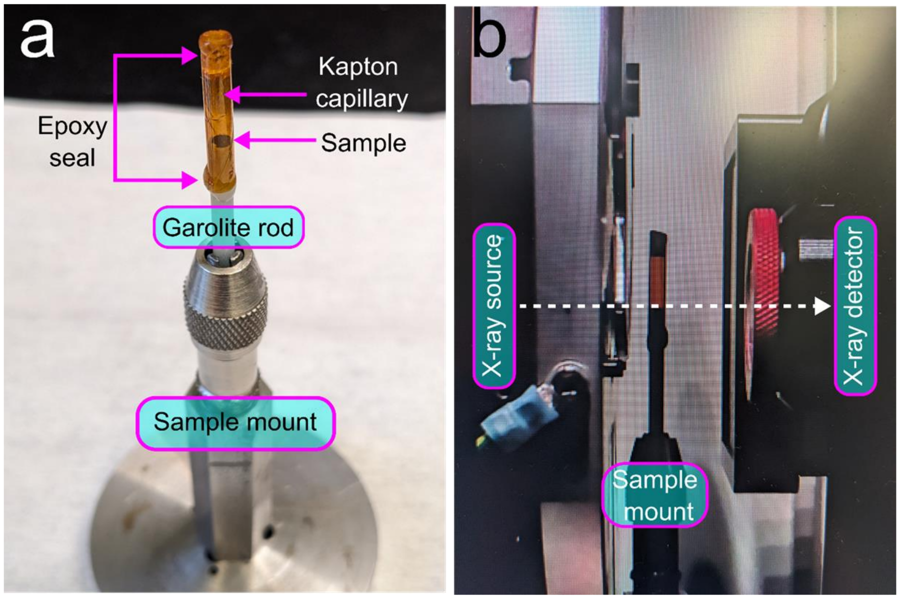

Figure S1. (a) Mounted sample for the microtomography scan (b) Experimental setup for the microtomography scan.

Table S1. X-ray micro-computed tomography experimental conditions.

Sample	Sample-to-source distance (mm)	Sample-to-detector distance (mm)	Exposire time (s)	Image pixel bin size
Pristine	11.1	19.5	20	Bin 2
300 cycles, 1C, 60 °C	10.2	16.5	7.5	Bin 4

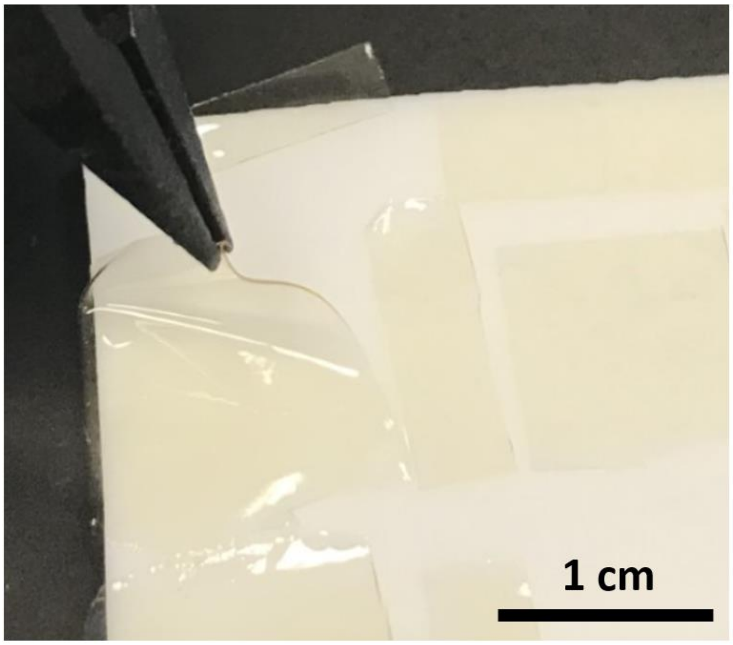

Figure S2. Photographs of the 4PGMA-PEG_{6k} SPE.

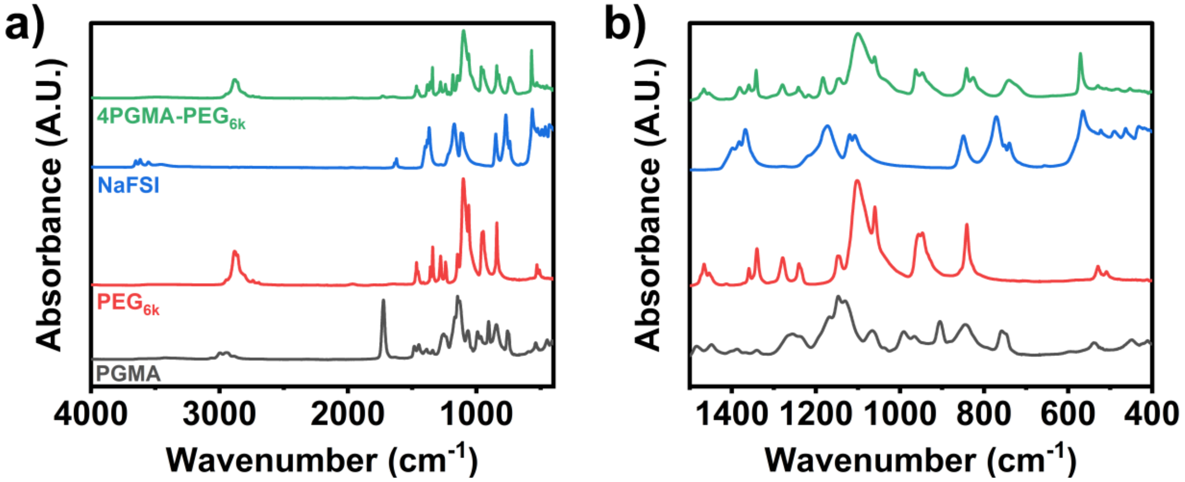

Figure S3. FTIR spectra from (a) 4000 to 400 cm^{-1} and (b) 1500 to 400 cm^{-1} for the 4PGMA-PEG_{6k} SPE, PGMA, PEG_{6k} and NaFSI salt.

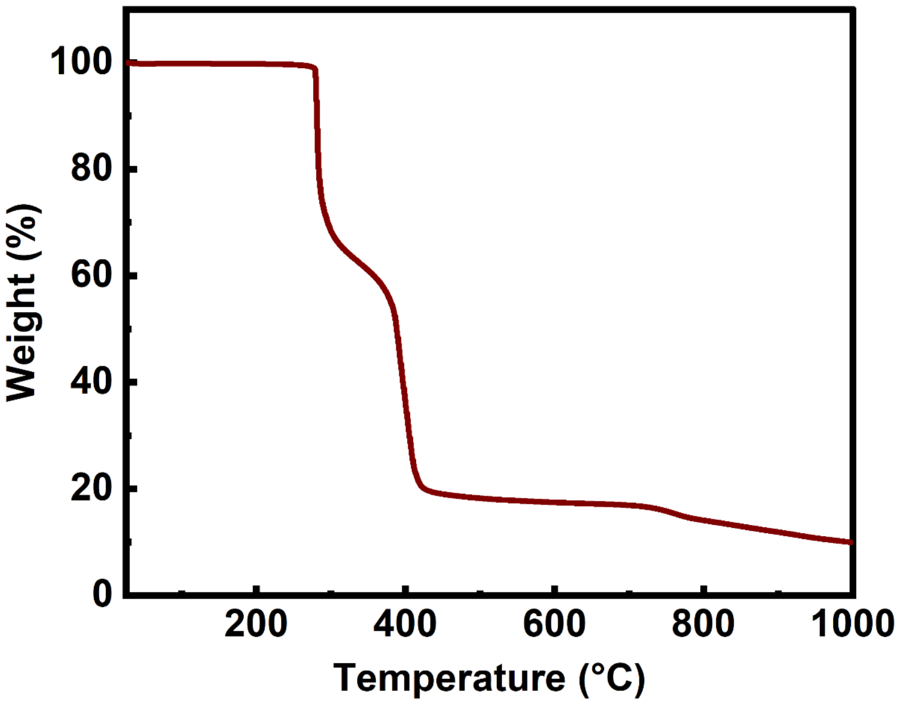

Figure S4. TGA scan of 4PGMA-PEG$_{6k}$ SPE.

Table S2. Thermal properties of 4PGMA-PEG_{6k} SPE, NNMO-CC and salt free monomers.

Sample	T_g (°C)	T_m (°C)	X_c (%)
4PGMA-PEG_{6k} SPE	-44.1	29.0	1.5
NNMO-CC	-38.8	29.3	10.0
PGMA	62	-	-
PEG_{6k}	-	60.9	-

Table S3. Mechanical properties of PGMA-PEG_{6k} SPE.

Young's modulus (MPa)	Tensile strength (MPa)	Elongation at break (%)	Toughness (MJ m^{-3})
1.5±0.1	1.5±0.1	181±3.7	1.6±0.1

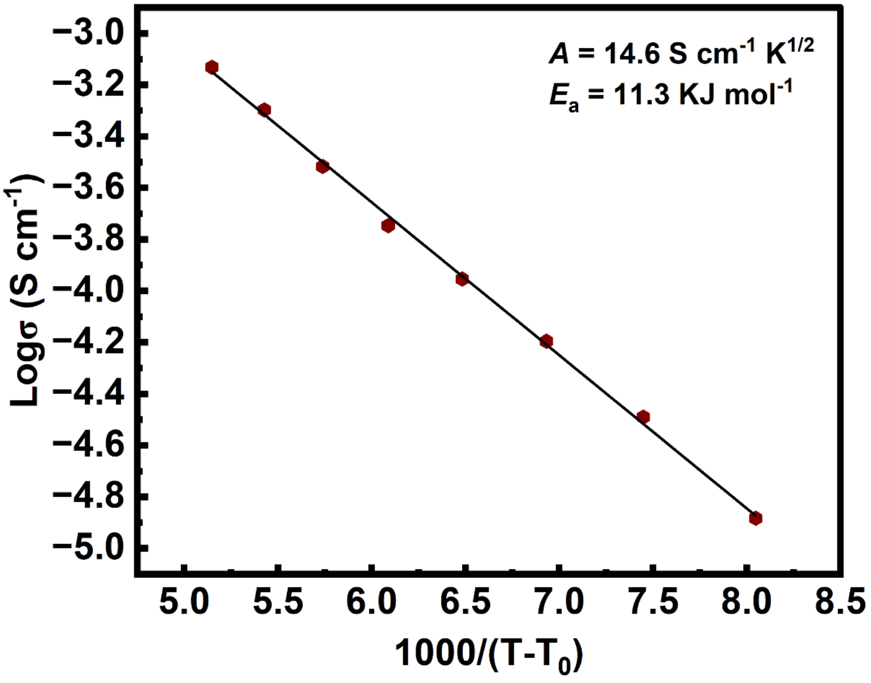

Figure S5. Linear VTF fit of the ionic conductivity of the 4PGMA-PEG$_{6k}$ SPE.

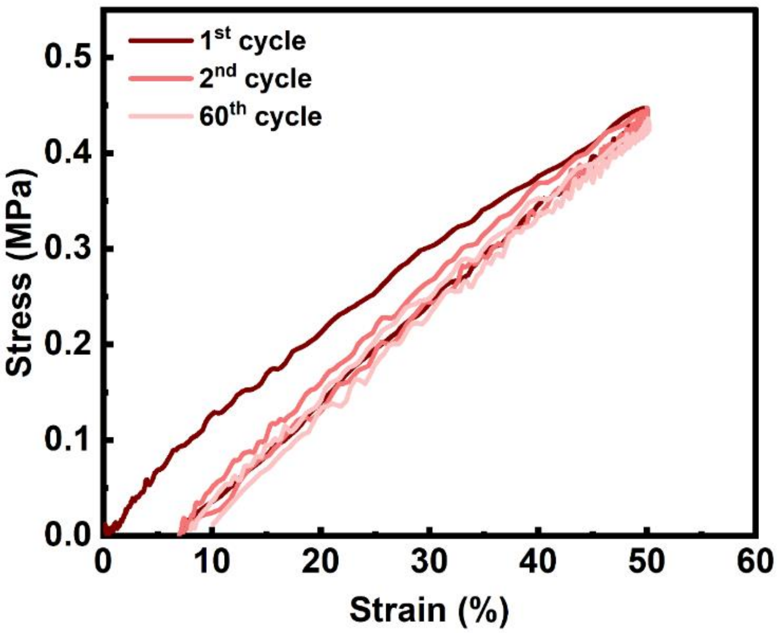

Figure S6. Strain cycling stress strain traces of 4PGMA-PEG_{6k} between 0 and 50% strain.

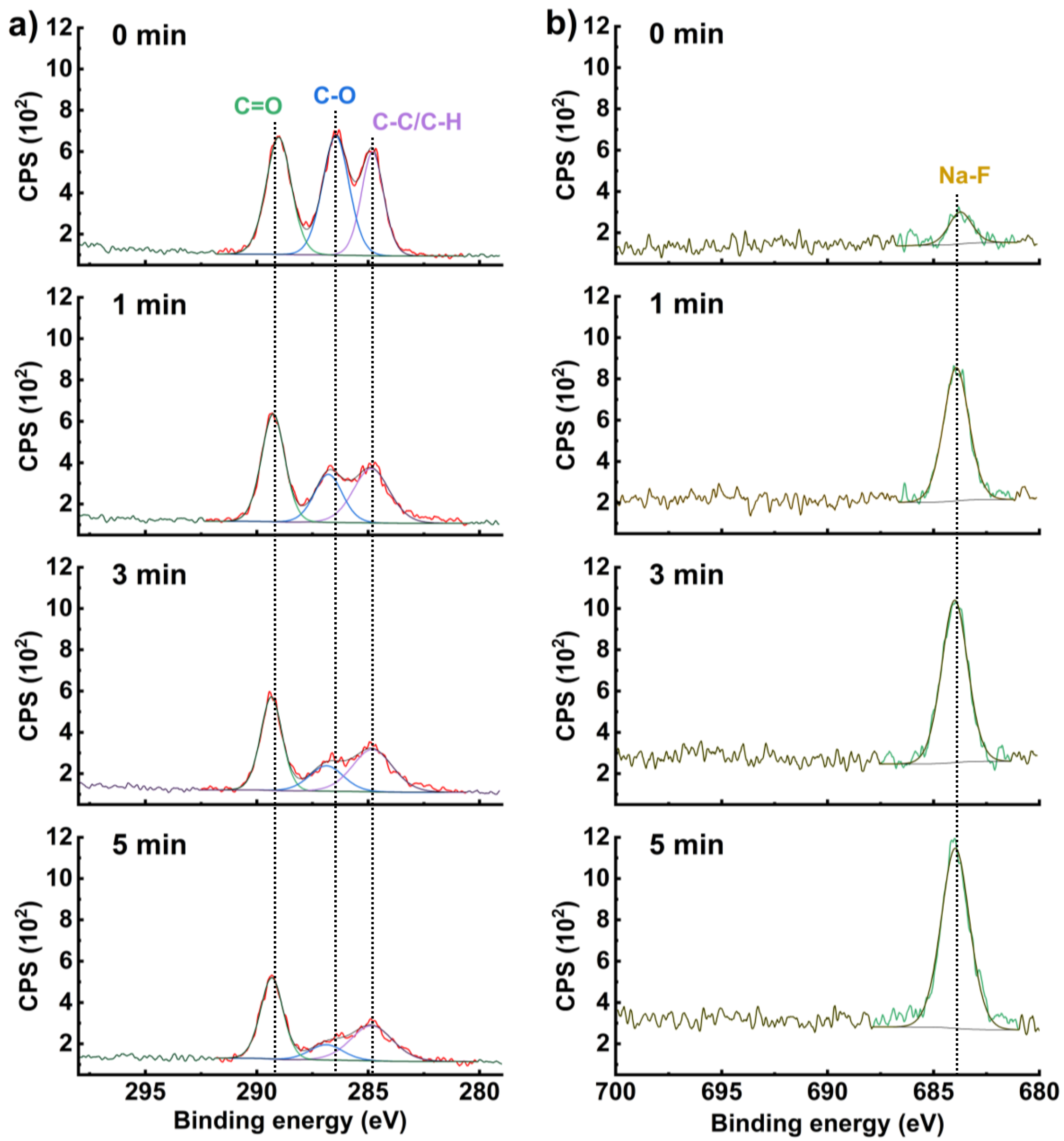

Figure S7. XPS spectra of (a) C1s and (b) F1s of cycled sodium symmetric cell anode surface in the pristine state and after 1, 3, and 5 mins of 2 kV Ar ion gun etching.

Table S4. Comparison of 4PGMA-PEG_{6k} symmetric cell performance to previously reported SEs in literature.

SPE/SCE	Temperature (°C)	Current density (mA cm^{-2})	Capacity (mAh cm^{-2})	Hours of continuous cyling (h)
**This work**	**80**	**0.5**	**1**	**4248**
PEO-CQDs-$NaClO_4$ [3]	60	0.2	0.5	120
PEO/$NaClO_4$/25Nasicon [4]	55	0.5	1	350
90PEO/$NaClO_4$/10Nasicon [5]	60	1	1	500
PEO/PVDF-HFP/$NaClO_4$/3D-NZSP [6]	25	0.1	0.1	700
Poly(EOm-b-PFPE) [7]	80	0.5	1	1000
P(EO)20/PEG-$NaClO_4$/20NZSP [8]	60	0.075	0.075	2500
POSS/PEG/$NaClO_4$ [9]	80	0.1, 0.5	0.25, 0.5	5100, 3500

Table S5. Comparison of 4PGMA-PEG_{6k} electrochemical performance to previously reported SEs in literature.

SPE/SCE-cathode	Temperature (°C)	Current density (mA cm^{-2})	Capacity (mAh cm^{-2})	Number of cycles	Mass loading (mg cm^{-2})	Capacity Loss
**This work**	**80**	**0.26 (1C)**	**0.2**	**700 1C, 80 °C**	**3**	**7.8%, 0.028%/cycle, 0.018 mAh g^{-1}/cycle, 1C, 80 °C**
PEO/NaFSI-$Na_{2/3}Ni_{1/3}Mn_{2/3}O_2$ [10]	80	0.2C	-	50	-	11%, 0.22%/cycle 0.12 mAh g^{-1}/cycle
PEO/NaFSI- $Na_{2/3}Ni_{1/3}Mn_{2/3}O_2$ [11]	60	0.033C	-	30	2.5	4.8%, 0.16%/cycle
PEGDMA-NaFSI-NVP [ℓ 12]	60	0.11 (0.5C)	0.22	720	1.9	9.9%, 0.014%/cycle
PEO-$NaPF_6$-NVP [13]	80	0.7 (2C)	0.35	200	3	14.2%, 0.071%/cycle
PMH9/P(VDF-HFP)/NaTFSI [14]	25	- (0.2C)	-	120	-	2%, 0.017% cycle
90PEO/$NaClO_4$/10Nasacon-$Na_3Zr_2Si_2PO_{12}$-NNMO[c, ℓ 4]	55	0.129-0.17 (0.5C)	0.26-0.34	100	3-4	1.6%, 0.016%/cycle
PEO/$NaClO_4$/25Nasicon-$Na_3Zr_2Si_2PO_{12}$[c 5]	60	0.19 (0.5C)	0.37	300	3	17%, 0.057%/cycle
PEO/NaTFSI/$Na_2Zn_2TeO_6$-NVP[c 15]	80	0.04 (0.2C)	0.21	100	2	6.6%, 0.066%/cycle

[ℓ] Contains liquid electrolyte.

[c] Composite polymer electrolyte.

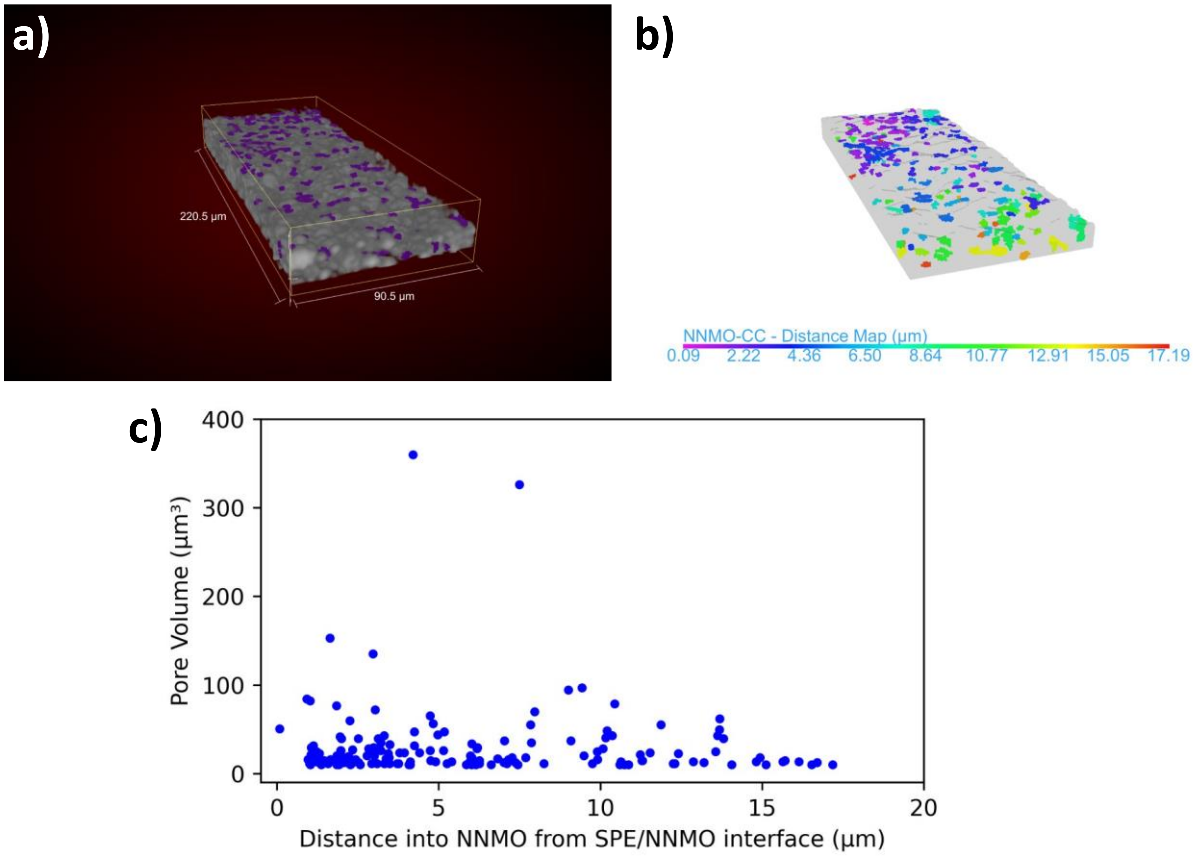

Figure S8. Micro-CT 3D reconstructions of pristine NNMO-CC illustrating (a) the spatial distribution of pores (purple) and (b) pore distribution as a function of depth into the cathode. (c) Quantitative analysis of pore size variation with depth into the cathode.

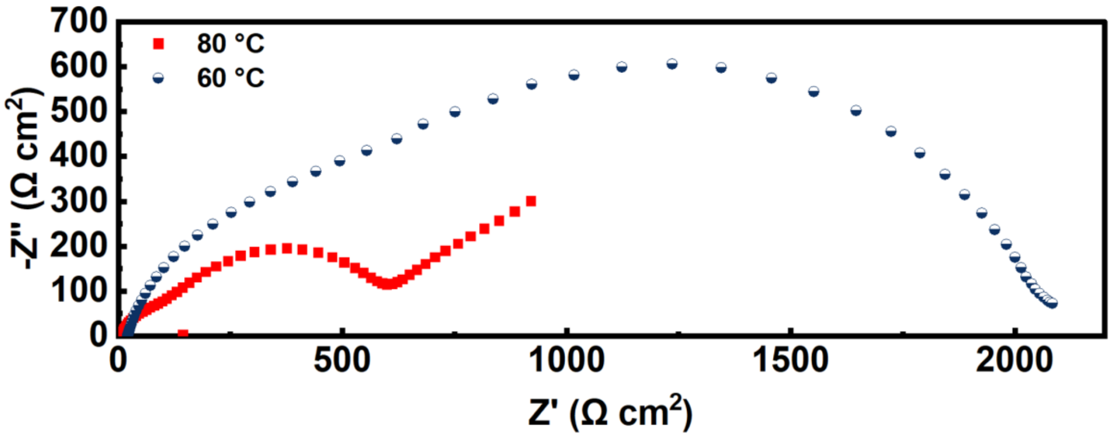

Figure S9. EIS spectra of Na|4PGMA-PEG_{6k}|NNMO-CC cell after pre cycling at 80 °C then lowering the temperature 60 °C prior to the rate capability experiment at 60 °C.

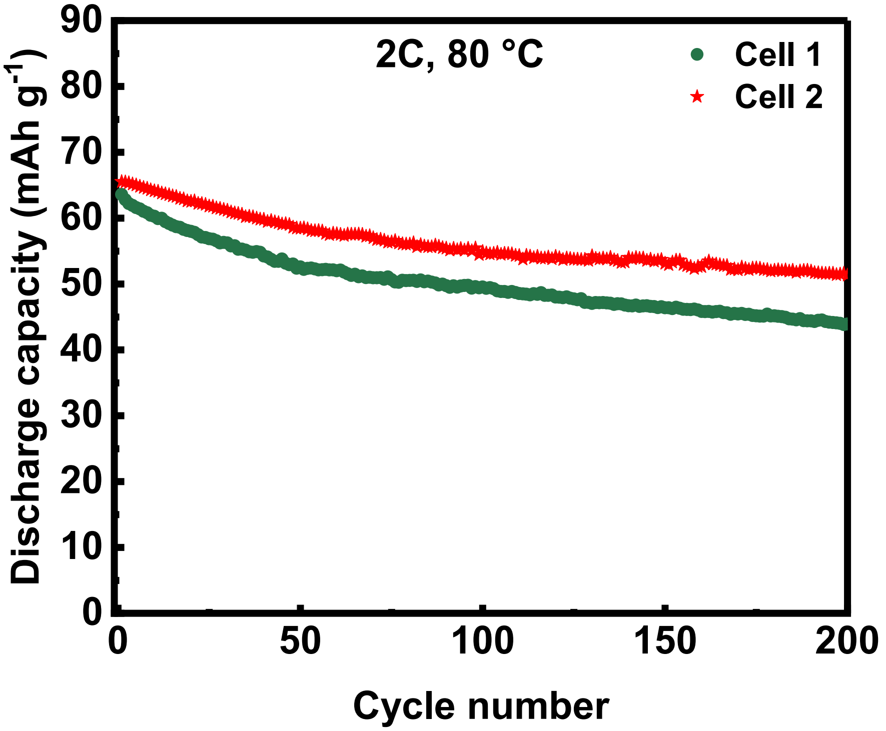

Figure S10. Discharge capacity vs cycle number for Na|4PGMA$_{6k}$|NNMO cells cycled at a rate of 2C at 80 °C.

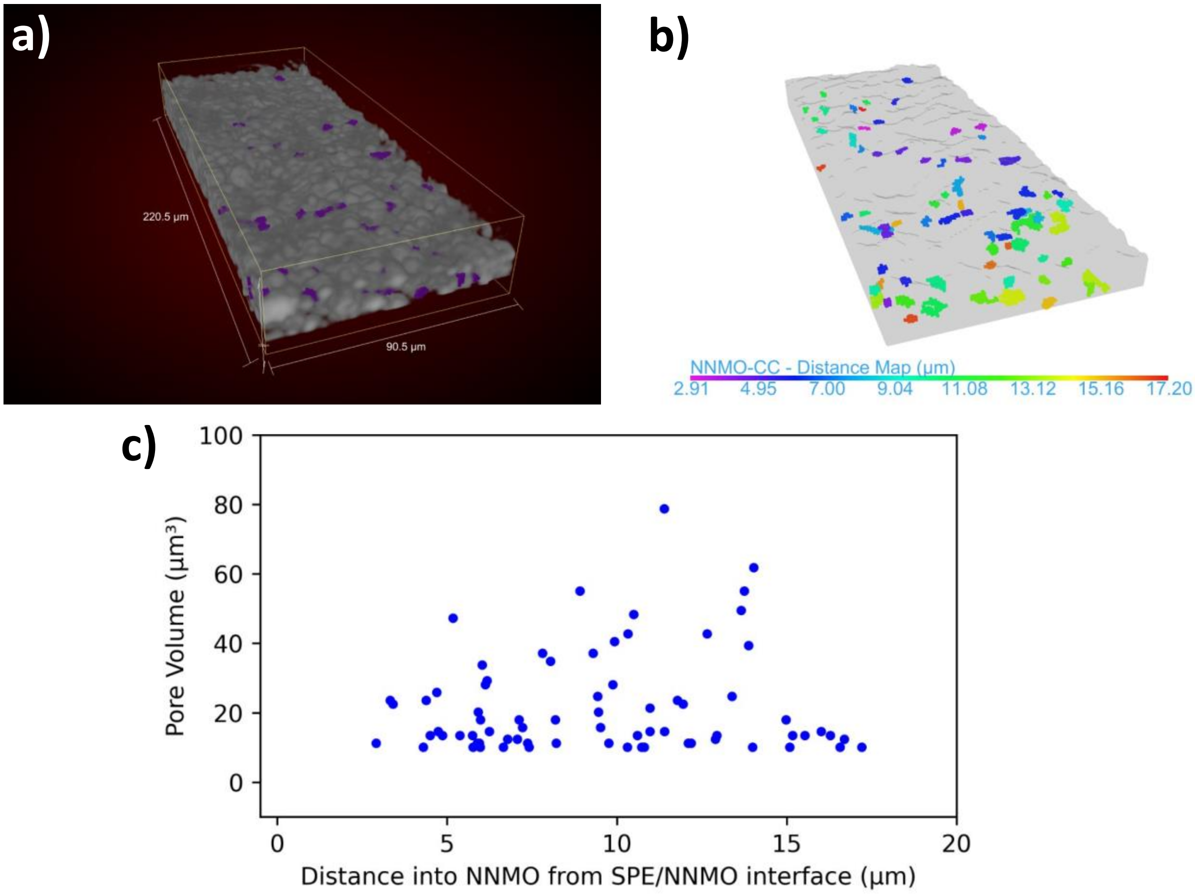

Figure S11 Micro-CT 3D reconstructions of NNMO-CC after 300 cycles at 60 °C illustrating (a) the spatial distribution of pores and (b) pore distribution as a function of depth into the cathode. (c) Quantitative analysis of pore size variation with depth into the cathode.

Table S6. Pore size and density for the pristine and cycled NNMO-CC.

Sample	Total pore density (#pores μm^{-3})	Pore density within 5 μm of SPE-NNMO-CC interface (#pores μm^{-3})	Average pore size (μm)
Pristine	5.21×10^{-4}	1.53×10^{-3}	30.1
300 cycles, 1C, 60 °C	1.65×10^{-4}	5.62×10^{-4}	38.1

References

(1) Kokaram, A. Practical, Unified, Motion and Missing Data Treatment in Degraded Video. *Journal of Mathematical Imaging and Vision* **2004**, *20* (1/2), 163-177. DOI: 10.1023/b:jmiv.0000011325.36760.1e.

(2) Ronneberger, O.; Fischer, P.; Brox, T. U-Net: Convolutional Networks for Biomedical Image Segmentation. Springer International Publishing, 2015; pp 234-241.

(3) Ma, C.; Dai, K.; Hou, H.; Ji, X.; Chen, L.; Ivey, D. G.; Wei, W. High Ion-Conducting Solid-State Composite Electrolytes with Carbon Quantum Dot Nanofillers. *Adv. Sci.* **2018**, *5* (5), 1700996. DOI: 10.1002/advs.201700996.

(4) Niu, W.; Chen, L.; Liu, Y.; Fan, L.-Z. All-solid-state sodium batteries enabled by flexible composite electrolytes and plastic-crystal interphase. *Chem. Eng. J.* **2020**, *384*, 123233. DOI: https://doi.org/10.1016/j.cej.2019.123233.

(5) Yu, X.; Xue, L.; Goodenough, J. B.; Manthiram, A. A High-Performance All-Solid-State Sodium Battery with a Poly(ethylene oxide)–Na3Zr2Si2PO12 Composite Electrolyte. *ACS Materials Letters* **2019**, *1* (1), 132-138. DOI: 10.1021/acsmaterialslett.9b00103.

(6) Wang, W.; Ding, M.; Chen, S.; Weng, J.; Zhang, P.; Yuan, W.; Bi, A.; Zhou, P. A novel composite solid electrolyte with ultrahigh ion transference number and stability for solid-state sodium metal batteries. *Chem. Eng. J.* **2024**, *491*, 151989. DOI: https://doi.org/10.1016/j.cej.2024.151989.

(7) Wang, X.; Zhang, C.; Sawczyk, M.; Sun, J.; Yuan, Q.; Chen, F.; Mendes, T. C.; Howlett, P. C.; Fu, C.; Wang, Y.; et al. Ultra-stable all-solid-state sodium metal batteries enabled by perfluoropolyether-based electrolytes. *Nat. Mater.* **2022**, *21* (9), 1057-1065. DOI: 10.1038/s41563-022-01296-0.

(8) Lanqing Zhao, M. H., Kun Ren, Dongrong Yang, Fupeng Li, Xiecheng Yang, Yingjie Zhou, Da Zhang, Shan Liu, Yong Lei, Feng Liang. Hot-Pressing Enhances Mechanical Strength of PEO Solid Polymer Electrolyte for All-Solid-State Sodium Metal Batteries. *Small Methods* **2024**, *8* (10).

(9) Zheng, Y.; Pan, Q.; Clites, M.; Byles, B. W.; Pomerantseva, E.; Li, C. Y. High-Capacity All-Solid-State Sodium Metal Battery with Hybrid Polymer Electrolytes. *Adv. Energy Mater.* **2018**, *8* (27), 1801885. DOI: 10.1002/aenm.201801885.

(10) Qi, X.; Ma, Q.; Liu, L.; Hu, Y. S.; Li, H.; Zhou, Z.; Huang, X.; Chen, L. Sodium Bis(fluorosulfonyl)imide/Poly(ethylene oxide) Polymer Electrolytes for Sodium-Ion Batteries. *ChemElectroChem* **2016**, *3* (11), 1741-1745. DOI: 10.1002/celc.201600221.

(11) Tatara, R.; Suzuki, H.; Hamada, M.; Kubota, K.; Kumakura, S.; Komaba, S. Application of P2-Na_{2/3}Ni_{1/3}Mn_{2/3}O₂ Electrode to All-Solid-State 3 V Sodium(-Ion) Polymer Batteries. *J. Phys. Chem. C* **2022**, *126* (48), 20226-20234. DOI: 10.1021/acs.jpcc.2c06360.

(12) Yao, Y.; Wei, Z.; Wang, H.; Huang, H.; Jiang, Y.; Wu, X.; Yao, X.; Wu, Z. S.; Yu, Y. Toward High Energy Density All Solid-State Sodium Batteries with Excellent Flexibility. *Adv. Energy Mater.* **2020**, *10* (12), 1903698. DOI: 10.1002/aenm.201903698.

(13) Zhang, Q.; Lu, Y.; Yu, H.; Yang, G.; Liu, Q.; Wang, Z.; Chen, L.; Hu, Y.-S. PEO-NaPF₆ Blended Polymer Electrolyte for Solid State Sodium Battery. *J. Electrochem. Soc.* **2020**, *167* (7), 070523. DOI: 10.1149/1945-7111/ab741b.

(14) Chen, G.; Ye, L.; Zhang, K.; Gao, M.; Lu, H.; Xu, H.; Bai, Y.; Wu, C. Hyperbranched polyether boosting ionic conductivity of polymer electrolytes for all-solid-state sodium ion batteries. *Chem. Eng. J.* **2020**, *394*, 124885. DOI: https://doi.org/10.1016/j.cej.2020.124885.

(15) Wu, J.-F.; Yu, Z.-Y.; Wang, Q.; Guo, X. High performance all-solid-state sodium batteries actualized by polyethylene oxide/Na2Zn2TeO6 composite solid electrolytes. *Energy Storage Mater.* **2020**, *24*, 467-471. DOI: https://doi.org/10.1016/j.ensm.2019.07.012.
